# Self-efficacy and positive coping mediate the relationship between social support and resilience in patients undergoing lung cancer treatment: A cross-sectional study

**DOI:** 10.3389/fpsyg.2022.953491

**Published:** 2022-09-23

**Authors:** Yizhen Yin, Mengmeng Lyu, Yiping Chen, Jie Zhang, Hui Li, Huiyuan Li, Guili Xia, Jingping Zhang

**Affiliations:** ^1^Xiang Ya Nursing School, Central South University, Changsha, Hunan, China; ^2^Alice Lee Centre for Nursing Studies, Yong Loo Lin School of Medicine, National University of Singapore, Singapore, Singapore; ^3^Shenzhen Hospital of Southern Medical University, Shenzhen, Guangdong, China; ^4^Hunan University of Chinese Medicine, Changsha, Hunan, China; ^5^Department of Orthopedics, West China Hospital, Sichuan University, Chengdu, Sichuan, China; ^6^Nethersole School of Nursing, Faculty of Medicine, The Chinese University of Hong Kong, Shatin, China

**Keywords:** lung cancer, resilience, self-efficacy, coping strategies, social support, structural equation modelling

## Abstract

**Background:**

The prognosis of patients undergoing lung cancer treatment might be influenced by mental health status. Resilience is one of the important predictors to reflect the mental health status. It has been shown that patients with higher levels of social support, self-care self-efficacy, and positive coping have greater resilience. This study aimed to determine the mediating role of self-efficacy and positive coping in the relationship between social support and psychological resilience in patients with lung cancer.

**Method:**

This is a cross-sectional study that was conducted in in the oncology departments and thoracic surgical wards of four tertiary hospitals in Hunan Province, China, between November 2016 and November 2017. Three hundred and three patients who were undergoing treatment for lung cancer volunteered their participation in the study. Participants completed questionnaires, including the Chinese version of the Perceived Social Support Seale Scale, the Chinese version of Strategies Used by People to Promote Health Scale, and the Chinese version of the Connor-Davidson Resilience Scale.

**Results:**

Mediation analysis indicated that self-care self-efficacy and social support partially mediate the effect of social support on resilience. Direct paths from social support to self-efficacy, self-efficacy to positive coping, positive coping to psychological resilience, self-efficacy to psychological resilience, and social support to psychological resilience were significant (*p* < 0.001). The indirect paths from social support to self-efficacy and self-efficacy to psychological resilience were also significant. The chain mediation from social support to self-efficacy, self-efficacy to positive coping, and positive coping to resilience were significant.

**Conclusion:**

Self-efficacy and positive coping play an important role in the relationship between social support and resilience in patients receiving cancer treatment. Social support not only directly influenced psychological resilience but also indirectly influenced psychological resilience through self-efficacy and positive coping.

## Introduction

Lung cancer is the second most common cancer and the leading cause of cancer-related death worldwide, accounting for about 11.4% of total cancer cases and 23% of all cancer deaths (Sung et al., 2021). Lung cancer is also the most frequently occurring cancer in China. In 2015, there were 787,000 newly diagnosed cases of lung cancer in China, accounting for 20% of total cancer diagnoses (Cao and Chen, 2019). Surgery and/or chemotherapy and radiotherapy is still the most effective treatment for early-stage lung cancer. However, treatment sides effects, expensive treatment costs, and uncertain treatment outcomes adversely affect the mental health status of the patients, leading to psychological distress such as depression, anxiety, and sleep disorder, which adversely affects patients’ quality of life, treatment compliance, and even immune system, and ultimately leading to increased length of hospital stay and poor prognosis (Yan et al., 2019; Bayley-Veloso et al., 2020; Arai et al., 2021; Rajapakse, 2021). Therefore, addressing the psychological concerns and improving their psychological wellbeing is of great importance in lung cancer patients who are undergoing treatment.

Despite experiencing higher psychological distress, those patients with higher levels of resilience experience lower levels of depression and sleep disorder and have better physical function and health-related outcomes (Hu et al., 2018; Lee and Jeong, 2019). Resilience is defined as the dynamic processes that an individual engages in to adapt successfully to stressors and adversities that threaten one’s function, development, or survival (Hinz, 1990). It is an important predictor of positive adaptation and psychological wellbeing. Cancer diagnosis and cancer treatment are considered traumatic life events for patients, resilience can buffer against psychological distress and improve patients’ quality of life during cancer treatment (Min et al., 2013; Wu et al., 2021). Therefore, the development of resilience intervention would of great importance in improving psychological wellbeing and health care outcomes in patients with lung cancer.

Understanding the factors that contribute to resilience is an important step in designing resilience interventions. The transactional model of resilience indicated that resilience is not static; the development process of resilience is driven by the interaction between adversities and protective factors (Richardson, 2002). If protective factors can resist the negative effects of adversities on individuals, resilience will maintain or increase, conversely, resilience will decrease. In the model, protective factors are classified into environmental factors (i.e., family, culture, community, school, and peers) and internal factors (i.e., cognitive, emotional, spiritual, physical, and behavioural factors; Richardson, 2002; Markovitz et al., 2015), indicating the surrounding environment or individual features may affect the development process of resilience (Tian et al., 2021a).

A systematic review of resilience showed that social support, coping, and self-efficacy are the main protective factors of resilience (Kim et al., 2019). Self-care self-efficacy is defined as one’s perceived confidence in addressing distress caused by cancer treatments and achieving positive health outcomes by controlling his or her behaviours and emotions (Wu et al., 2021). Patients with higher levels of self-care self-efficacy have more confidence in their ability to fight against disease and manage the side effects of cancer treatments (Papadopoulou et al., 2017; Phillips et al., 2017). Previous studies have demonstrated that self-care self-efficacy can promote resilience and reduce cancer-related psychological distress (Papadopoulou et al., 2017; Phillips et al., 2017). Social support refers to perceived psychological, material, and informational support from family, friends, or others such as healthcare providers (Tian et al., 2021b). Many studies found that higher levels of social support were associated with higher levels of resilience in oncology patients (Liu et al., 2018; Tian et al., 2021a). Coping refers to the process by which an individual adopts different coping strategies to manage problems and psychological distress resulting from stressful situations (Darabos et al., 2021). Studies have shown that positive coping was associated with higher levels of resilience (van de Wiel et al., 2021; Wu et al., 2021). Although studies identified the relationships between resilience and these social, cognitive, and behavioural factors, the mechanism underlying the interactions between these factors and the degree of the influence of these factors on resilience in patients undergoing lung cancer treatment remains unclear.

**Figure 1:**
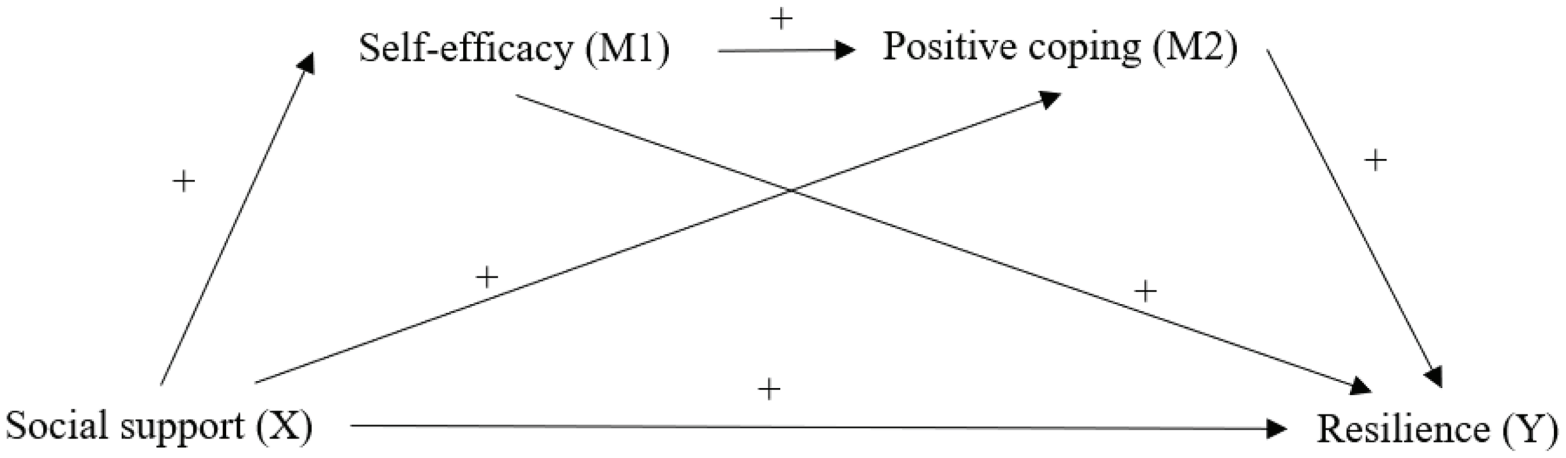
The hypothetical mediation model of positive coping and self-care self-efficacy mediated the relationships between social support and resilience.

According to Bandura’s self-efficacy theory, social support can promote self-efficacy through verbal encouragement. Previous studies supported that higher social support was associated with higher levels of self-efficacy in cancer patients (Shen et al., 2020; Lingens et al., 2021). Lazarus and Folkman’s stress and coping theory describe that individual manages stress in different ways, which is in part determined by patients’ evaluation of their abilities and resources in addressing problems caused by stressful situations (Darabos et al., 2021). The basic assumption of the model is that the perception of a lack of resources leads to the utilization of negative coping strategies such as denial and avoidance. Conversely, individuals with positive attitudes towards the stressful situation and who have more confidence in problem-solving use more positive coping strategies, suggesting higher levels of self-efficacy were associated with more positive coping strategies (Chirico et al., 2017; Wu et al., 2021). Besides, studies on inpatients found that higher levels of social support were correlated with more positive coping strategies (Grant et al., 2013; Kang et al., 2020). Based on the theories and the literature, this study hypothesized that self-efficacy and positive coping mediated the relationship between social support and psychological resilience in patients who are undergoing treatment for lung cancer (Figure 1).

This study aimed to examine the mediating effect of positive coping and self-care self-efficacy on the relationship between social support and resilience among patients undergoing lung cancer treatment. Findings of this study would contribute to the understanding of the role of social support in improving resilience in patients receiving lung cancer treatments and provide oncologists and oncology nurses information on designing resilience interventions for lung cancer patients who undergoing treatment, to improve social resources, confidence, and ability to adapt to cancer diagnosis and cancer treatments.

## Materials and methods

### Participants and procedure

The cross-sectional study was conducted in four tertiary hospitals in Hunan Province, China. Convenience sampling was used to select patients who were undergoing treatment for lung cancer in the oncology departments and thoracic surgical wards of the hospital between November 2016 and November 2017 (Jobst et al., 2021). Patients were included, if they: (1) were diagnosed with lung cancer by pathological or cytological examination, (2) were receiving treatment for lung cancer, (3) aged 18 years old and above, (4) were proficient in reading and writing Mandarin, and (5) expressed willingness in participant in the study. Participants were excluded, if they: (1) were diagnosed with mental illness and cognitive impairment, (2) were diagnosed with other types of cancer or life-threatening diseases, and (3) were engaging in other resilience-related studies. According to the principle of the structural equation model, the basic sample size is 200, with adding 20 participants for each estimated parameter in the model (Jobst et al., 2021). In the hypothesized model, four estimated parameters were included, thus the minimum sample size of this study should be 280. Taking the dropout rate as 10%, the minimum sample should be 308.

This study was approved by the Institutional Review Board at the Central South University (approval number: 2016038). Two trained oncology nurses of each hospital reviewed the electronic medical record system and approached patients who met the inclusion and exclusion criteria. Five hundred and ninety-four eligible patients were approached. The nurses explained the aim, content, process, and potential risks and benefits of the study to the patients and answered all their queries to ensure they fully understand the study. A total of 320 participants signed informed consent and completed the paper-version questionnaire independently in the hospitals. Three hundred and three valid questionnaires were recovered, with 17 questionnaires being excluded due to over 20% of missing information.

## Measures

### Demographic and clinical information

The demographic and medical characteristics of the participants were collected using a self-compiled questionnaire that included age, sex, education level, monthly income, and pathological classification.

### Perceived social support scale (PSSS)

Social support was assessed with the 12-item measure developed by Zimet (Zhou et al., 2015). The PSSS consisted of three domains, measuring support from family, friends, and others (health care providers). Each item is scored on a seven-point Likert scale from 1 (strongly disagree) to 7 (strongly agree). Scores can range from 12 to 84, with higher scores reflecting higher levels of perceived social support. The Chinese version of the PSSS has good internal reliability (Zhou et al., 2015). In this study, Cronbach’s α was 0.915.

### Strategies used by people to promote health (SUPPH)

Self-efficacy specific to individuals experiencing cancer treatment was assessed by the 29-item SUPPH developed by Lev (Yuan et al., 2015). The scale includes three subscales: positive attitude, stress reduction, and making decisions. Each item is scored on a five-point Likert scale from 1 (unconfident) to 5 (very confident). Scores can range from 29 to 145, with higher scores indicating higher levels of confidence in coping with cancer treatment. Yuan et al. examined the Cronbach’s α of the Chinese version of the SUPPH (0.849–0.970) (Yuan et al., 2015). In the present study, Cronbach’s α was 0.971.

### Connor-Davidson resilience scale (CD-RISC)

Resilience was measured using the Chinese version of the CD-RISC first developed by Kathryn M. Connor and Jonathan R.T and translated by Yu et al. (Wu et al., 2017). The 25-item self-reporting scale has three domains, including tenacity, strength, and optimism. Each item is scored on a five-point Likert scale ranging from 0 (not true at all) to 4 (often true). These ratings result in a total score ranging from 0 to 100, with higher scores reflecting higher resilience. The Chinese version of the CD-RISC scale showed good reliability (0.91; Wu et al., 2017). In this study, Cronbach’s α was 0.937.

### Medical coping modes questionnaire (MCMQ)

Positive coping strategies were measured using the confrontation subscale of the MCMQ (Shen and Jiang, 2000). The confrontation subscale includes eight items corresponding to actively looking for disease-related information and instrumental support coping (e.g., “How much do you know about your illness from books, magazines, and newspapers in recent months?”). Each item is measured with a four-point Likert scale ranging from 1 (strongly disagree) to 5 (strongly agree). The Chinese version of the MCMQ scale showed good reliability (Shen and Jiang, 2000). In the present study, Cronbach’s α of confrontation subscale was 0.86.

### Statistical analysis

The descriptive statistics of the participants will be evaluated to summarize demographic and clinical characteristics. The Student’s t-test and one-way ANOVA were used to compare the CD-RISC total scores between participants with different demographic and clinical characteristics. Pearson’s correlation was carried out to examine the correlations between resilience, social support, self-care self-efficacy, and positive coping strategies. Hierarchical regression analysis was used to analyse the relationships between resilience and social support, self-care self-efficacy, and confrontation coping. All tests of significance were two-tailed and *p* < 0.05 was considered statistically significant. SPSS version 18.0 was used for conducting descriptive statistics and Pearson’s correlation.

Second, structural equation modelling (SEM) was computed with AMOS 17.0 (IBM Corporation, Armonk, NY, United States) to test the hypothesized model with the maximum likelihood estimation methods. The input for each analysis was the covariance matrix of the items. The goodness-of-fit of the model was evaluated using absolute and relative indices. The relative indices are the *χ*^2^/*df*, Goodness-of-Fit Index (GFI), adjusted GFI (aGFI), Normed Fit Index (NFI), Incremental Fit Index (IFI), Tacker-Lewis Index (TLI), and Comparative Fit Index (CFI). Relative fit index values greater than.90 indicate a good model fit (Hoyle, 1995). The absolute index is the Root Mean Square Error of Approximation (RMSEA). As a rule of thumb, values near.05 for RMSEA indicate a good model fit (Browne and Cudeck, 1992).

## Results

### Sample characteristics and associations with resilience

As shown in Table 1, the mean (standard deviation [SD]) age of the 303 subjects was 56.74 (10.93) years, and 78.5% of them were aged 50 years old and above, 64.7% were male, 95.4% were married, 36.4% were junior high school graduates, 72.9% reported monthly household income less than 3,000 RMB (444.24 USD, which is lower than the Chinese household disposable income *per capita*), 29.4% were diagnosed with stage IV lung cancer, 38.3% were diagnosed with adenocarcinoma, and 38.0% received surgery only. The results of t-test and analysis of variance showed that there were significant differences in resilience score between different genders (*t* = 2.36*, p* = 0.019), education level (*F* = 13.81, *p* = 0.000), monthly income (*F* = 11.99, *p* = 0.000), and pathological classification (*F* = 2.62, *p* = 0.035). The mean scores (SD) of the social support, self-care self-efficacy, confrontation coping and resilience and the correlations between these variables were shown in Table 2.

**Table 1:** Demographic and clinical characteristics and univariate associations with resilience.

		*N* (%)	CD-RISC		
			Mean (SD)	*t*/*F*	*p*
Gender	Male	196 (64.7)	51.53 (15.02)	2.36	0.019^*^
	Female	107 (35.3)	47.23 (15.35)		
Age (years old)	< 30	4 (1.3)	48.50 (13.23)	0.05	0.996
	30–39	15 (5.0)	51.33 (12.21)		
	40–49	46 (15.2)	50.09 (15.87)		
	50–59	107 (35.3)	49.78 (15.30)		
	≥ 60	131 (43.2)	50.07 (15.55)		
Marital status	Single	8 (2.6)	55.25 (10.51)	1.62	0.199
	Married	289 (95.4)	52.06 (15.93)		
	Else (widowed or divorced)	6 (2.0)	63.33 (17.49)		
Education level	Primary school and above	90 (29.7)	45.91 (13.31)	13.81	0.000^*^
	Junior high school	105 (34.6)	46.45 (14.03)		
	Senior high school	76 (25.1)	55.37 (16.64)		
	College/university	32 (10.6)	60.50 (12.26)		
Monthly income (RMB)	< 3,000	221 (72.9)	47.5 (14.59)	11.99	0.000^*^
	3,000–4,999	59 (19.5)	56.22 (12.53)		
	≥ 5,000	23 (7.6)	58.17 (20.33)		
Stages of cancer	Stage I	54 (17.8)	51.47 (18.55)	1.48	0.209
	Stage II	57 (18.8)	56.60 (15.79)		
	Stage III	54 (17.8)	50.46 (13.52)		
	Stage IV	89 (29.4)	52.52 (15.08)		
	Unclear	49 (16.2)	50.25 (16.39)		
Cancer treatment	Surgery	115 (38.0)	54.80 (16.96)	1.66	0.131
	Chemotherapy or radiotherapy	38 (12.5)	48.24 (14.68)		
	Chemotherapy and radiotherapy	83 (27.4)	52.90 (13.31)		
	Surgery and chemotherapy or radiotherapy	29 (9.6)	55.15 (15.74)		
	Other (Chinese medicine)	38 (12.5)	50.03 (18.89)		
Pathological Classification	Squamous carcinoma	98 (32.3)	53.47 (15.13)	2.62	0.035^*^
	Adenocarcinoma	116 (38.3)	48.52 (14.70)		
	Small cell carcinoma	27 (8.9)	44.85 (14.13)		
	Other	27 (8.9)	47.41 (14.43)		
	Unsure	35 (11.6)	51.26 (15.25)		

* *p* < 0.05;

** *p* < 0.01.

**Table 2:** Correlations among the measured variables.

Variables	Mean (SD)	Social support	Self-care self-efficacy	Confrontation coping	Resilience
Social support	60.95 (10.30)	1			
Self-care self-efficacy	81.38 (20.22)	0.272^*^	1		
Confrontation coping	19.47 (2.68)	0.151^*^	0.174^*^	1	
Resilience	52.37 (15.88)	0.364^*^	0.599^*^	0.246^*^	1

* *p* < 0.05;

** *p* < 0.01.

### Hierarchical regression results with resilience as the dependent variable

Hierarchical regression analysis was used to analyse the relationships between resilience and social support, self-care self-efficacy, and confrontation coping. Univariate analyses identified that sex, education level, monthly income, and pathological classification are associated with resilience. Therefore, these demographic characteristics were selected as covariates. As shown in Regression Model 1 (Table 3), after controlling covariates, social support (*β* = 0.297, *p* = 0.000) had significant effects on resilience. In Regression Model 2, with resilience as the dependent variable, linear regression found that the Beta of social support decreased but was still significant after adding self-care self-efficacy as an independent variable. In Regression Model 3, with resilience as the dependent variable, linear regression found that Beta of social support and self-care self-efficacy decreased but were still significant after adding coping as an independent variable. This finding suggests that self-efficacy partly mediates the relationship between resilience and social support while coping partly mediates the relationships between resilience and social support and self-care self-efficacy, supporting the hypothesized model (Figure 1).

**Table 3:** Summary of hierarchical regression analysis for variables predicting resilience.

	Model 1			Model 2			Model 3		
	Beta	*t*	*p*-value	Beta	*t*	*p*-value	Beta	t	*p*-value
(Constant)	-	2.640	0.009		−0.248	0.804		−1.789	0.075
Pathological Classification	−0.023	−0.453	0.651	−0.042	−0.960	0.338	−0.036	−0.825	0.410
Gender	−0.084	−1.671	0.096	−0.054	−1.241	0.216	−0.058	−1.348	0.179
Education level	0.196	3.608	0.000	0.113	2.407	0.017	0.112	2.402	0.017
Monthly income	0.211	3.831	0.000	0.150	3.179	0.002	0.145	3.088	0.002
Social support	0.297	5.804	0.000	0.185	4.129	0.000	0.174	3.895	0.000
Self-care self-efficacy	-	-	-	0.489	10.615	0.000	0.474	10.292	0.000
Coping	-	-	-	-	-	-	0.111	2.557	0.011
Adjusted *R*^2^	0.242			0.450			0.460		
Δ*R*^2^	-			0.206			0.012		
Δ*F* – value	-			112.69			6.54		
*p*-value	-			0.000			0.011		

### The structural equation model analysis

**Figure 2:**
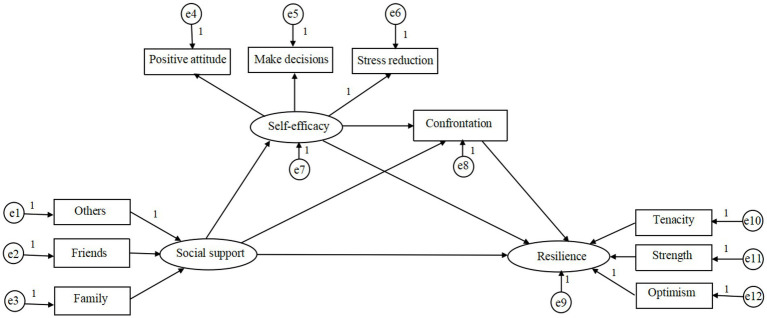
The original model. e1–e6 and e10–e12 are the measurement errors of each observed variable to estimate latent variables; e7–e9 are the residuals that may affect the endogenous latent variables except the exogenous latent variables.

**Figure 3:**
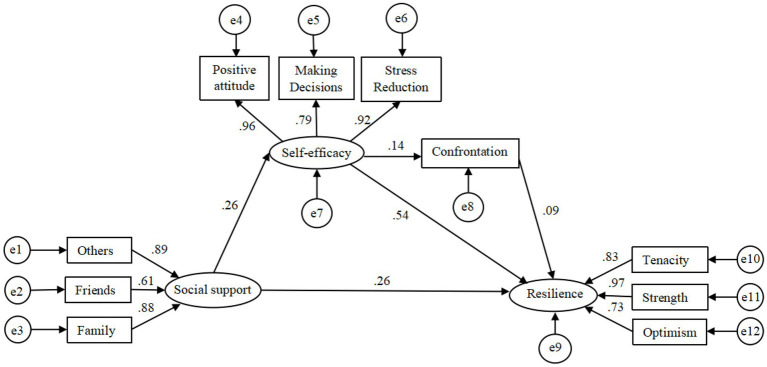
The modified structural equation model on the relationship between social support, self-efficacy, positive coping, and psychological resilience.

The author firstly constructed the structural equation model based on the hypothesized model (Figure 1). Maximum likelihood method was used to estimate parameters and built the structural equation model (SEM). The original model showed an unsatisfactory fit (Figure 2). Then, the model was modified based on modification indices. The nonsignificant pathway between social support and coping was removed (*p* = 0.487). Based on the modification indices, the correlations between some residuals of the observed variables should be added, such as e5 ↔ e10, e3 ↔ e6, and so on. However, the added parameter relationships cannot violate the SEM assumptions that: (1) residuals of observed variables are not correlated with latent variables (no covariance can be established) and (2) residuals of the observed variables can have covariate relationships but cannot have path causality. After removing nonsignificant path and adding covariation, we obtained the modified model (Figure 3). The model indicated good fit, *χ*^2^/*df* = 34.7/27 = 1.28 < 2, GFI = 0.978, aGFI = 0.956, CFI = 0.996, IFI = 0.996, TLI = 0.993, NFI = 0.982, RMSEA = 0.031. Corresponding numerical results were summarized in Table 4; Figure 3. As illustrated, social support (*β* = 0.026, *p* = 0.001), self-care self-efficacy (*β* = 0.54, *p* = 0.001), and positive coping (*β* = 0.09, *p* = 0.041) had significant effects on resilience. Social support (*β* = 0.145, *p* = 0.001) and self-care self-efficacy (*β* = 0.013, *p* = 0.049) had significant indirect effects on resilience.

**Table 4:** Decomposition of standardized effects from the model.

Variables	Social support			Self-care self-efficacy		Positive coping
	Self-care self-efficacy	Positive coping	Resilience	Positive coping	Resilience	Resilience
Total effects	0.262^**^	0.037^*^	0.403^**^	0.142^*^	0.552^**^	0.093^*^
Direct effects	0.262^**^	-	0.258^**^	0.142^*^	0.539^**^	0.093^*^
Indirect effects	-	0.037^*^	0.145^**^	-	0.013^*^	-

* *p* < 0.05;

** *p* < 0.01.

## Discussion

This study aimed to explore the role of self-efficacy and positive coping in the relationship between social support and resilience in patients undergoing lung cancer treatments. The results indicated that self-efficacy and positive coping were positively correlated with social support and psychological resilience. Self-efficacy and positive coping had a partial mediating role in the relationship between social support and resilience. The results partially supported our hypothesis.

### The relationship between social support, self-efficacy, positive coping, and psychological resilience

The direct effects of social support, self-care self-efficacy, and positive coping on resilience were identified, which is consistent with the findings of previous studies, indicating that social support, self-efficacy, and positive coping were significant predictors of resilience (Hu et al., 2018; Liu et al., 2018; Wu et al., 2021). The results also echoed the finding of a qualitative study. Li et al. (2020) demonstrated that good social support, excellent psychological qualities, and self-care activities contributed to the resilience process in patients with lung cancer. These findings supported the hypotheses proposed by the transactional model of resilience: both environmental and internal factors could increase resilience by buffering the adverse impact of stressors on individuals (Richardson, 2002; Markovitz et al., 2015). Social support is beneficial for improving resilience through emotional and material support. A qualitative study found that information, emotion, financial, and material supports from family, friends and fellow patients in cancer wards are of great importance in promoting adaption to cancer and its treatments (Hauken and Larsen, 2019). A study in 272 cancer patients and their spouses also indicated that perceived social support from spouses and other patients was associated with higher levels of individual resilience in cancer patients (Chen et al., 2021). The direct effect of self-care self-efficacy on resilience was also significant—the higher perceived self-efficacy in coping with treatment-related side-effects and psychological distress, the higher levels of resilience. Patients with higher levels of self-care self-efficacy have more confidence in regulating their emotions, cognition, and behaviour to achieve positive health outcomes, which can promote resilience processes through activating emotional, motivational, and behavioural mechanisms aimed at actively responding to stressors (Wu et al., 2021). Consistent with previous studies, in this study, positive coping was found to be associated with higher levels of resilience (Grant et al., 2013; Darabos et al., 2021). In the current study, positive coping is a confrontation coping style that adapts to cancer by getting advice from others and asking for help to reduce the treatment side effects and associated psychological distress. Previous studies found that lower levels of psychological distress and fewer treatment side effects were associated with higher levels of resilience (Hou and Lam, 2014; Zhang et al., 2018). These findings support that the development process of resilience is affected by environmental and individual features.

### The mediating role of self-efficacy and positive coping

As expected, self-care self-efficacy played a mediating role in the relationship between social support and resilience. Based on the present results and Bandura’s self-efficacy theory, supportive family, friends, health care providers and fellow cancer patients suggest new and positive perspectives on cancer, provide information on self-care and symptom management and encourage patients to adapt to their situation, which may improve patients’ confidence in their ability in coping with cancer treatments (Liu et al., 2020). When lung cancer patients fail to get sufficient support, they feel are left to cope with cancer alone, which can reduce self-care self-efficacy (Chan et al., 2020). The decreased self-care self-efficacy may affect one’s coping style in managing stressful situations. In the current study, positive coping severed as a mediator in the association between self-efficacy and resilience, supporting the hypothesis. According to Lazarus and Folkman’s stress and coping theory, the mechanism underlying the effects of self-efficacy on coping is that individuals with higher levels of self-efficacy have more confidence in regulating their behaviour or emotion to cope with stressors and thus have a higher tendency to participate in activities to combat stressful situations (Darabos et al., 2021). Cancer patients with high self-efficacy expectations focus more on the demands of stressful situations and feel more efficacious in their ability in coping with changes and challenges resulting from cancer, which can promote better psychological adjustment and higher levels of resilience (Freire et al., 2020). Studies on cancer patients supported that a higher level of self-efficacy was associated with more self-care behaviours and positive coping strategies, such as relaxation, cognitive restructuring, and assertive communication (Johansson et al., 2018; Chou, 2019; Jacobs et al., 2020). It has been found that as the positive coping strategies increased, psychosocial adjustment also improved, which add to increased resilience (Yu et al., 2022).

The pathway between social support and positive coping was not significant, which was contrary to previous studies (Grant et al., 2013; Kang et al., 2020). However, the indirect effect of social support on positive coping *via* the mediating effect of self-care self-efficacy was significant. This finding was consistent with previous studies, demonstrating that perceived social support can promote the utilization of positive and effective coping skills by enhancing more positive self-appraisal (Labrague, 2021; Li et al., 2021). According to the illness self-regulation model explicates, the non-significant direct relationship between the variables may be explained as follows. Disease-related coping strategies were determined by an individual’s perceptions of risk of illness, perceived ability in controlling disease progression, and negative emotional arousal experience in facing illness threats (Leventhal et al., 2016). Social support as a contextual factor can affect patients’ perceptions of illness threats and relieve psychological distress, while it has no direct effects on one’s coping strategies (Kang et al., 2020). However, future studies are needed to further explore the mechanism underlying the effects of social support on coping strategies in cancer patients.

### Clinical implications

This study suggested that oncology health care professionals should pay more attention to resilience in patients undergoing treatment for lung cancer through regularly evaluating resilience and providing resilience intervention for those with low levels of resilience. A systematic review on interventions to promote resilience in cancer patients (Ludolph et al., 2019). A significant positive effect on resilience was achieved by interventions based on cognitive behavioural therapy, mindfulness-based psychotherapy, and supportive-expressive group therapy. However, fewer interventions were developed based on resilience-related theories. Theories could help health care providers to identify intended goals of the intervention, which may increase intervention effectiveness (O'Cathain et al., 2019). This study examined factors associated with the development process of resilience based on the transactional model of resilience, which identified the potential targets of intervention for improving resilience. Given the direct and indirect effects of social support, self-care self-efficacy and positive coping on resilience, resilience interventions for lung cancer patients should be developed to simultaneously increase social support, self-care self-efficacy and positive coping.

### Study limitations

There are some limitations to this study. First, resilience was measured using CD-RISC. Although the scale has been administered in studies assessing oncology patients’ resilience and demonstrated good reliability (Solano et al., 2016; Sharpley et al., 2017; Arrato et al., 2022), this scale is a generic-based resilience measure that may lack sensitivity to tap dimensions of resilience in patients with cancer. Further studies are needed to develop resilience scales targeting cancer patients. Second, the current study is a cross-sectional study, even if the SEM approach demonstrated the direct and indirect associations of included variables, these should not be interpreted as causality. Further longitudinal studies or experimental studies are warranted to identify the causalities among the variables. Third, clinical factors, such as time since diagnosis, cancer-related symptoms, treatment side-effects and physical function, that may affect patients’ resilience were not included in the current study. Therefore, studies should further explore various factors that may affect psychological resilience in patients undergoing cancer treatment. Finally, there are geographical and cultural limitations of the study sample. Combining historical, geographical, ethnic and cultural dimensions, China can be divided into seven geographical divisions: East China, South China, North China, Central China, Southwest China, Northwest China and Northeast China. Medical, economic, cultural and educational levels vary widely across regions in China. In this study, the study sample was selected only from a province located in Central China, which limits the generalizability of the study results.

## Conclusion

The present study provides preliminary evidence of the mediating role of self-care self-efficacy and social support in the relationship between social support and resilience. Therefore, in the future, to develop interventions for promoting psychological resilience among lung cancer patients, these findings should be taken into consideration.

## Data availability statement

The original contributions presented in the study are included in the article/Supplementary material, further inquiries can be directed to the corresponding author.

## Ethics statement

The studies involving human participants were reviewed and approved by Institutional Review Board at the Central South University (approval number: 2016038) approved this study. The patients/participants provided their written informed consent to participate in this study.

## Author contributions

YY and ML: performed the material preparation, data collection, analysis and wrote the first draft of the manuscript. All authors contributed to the study’s conception, design, and commented on the previous versions of the manuscript. All authors contributed to the article and approved the submitted version.

## Conflict of interest

The authors declare that the research was conducted in the absence of any commercial or financial relationships that could be construed as a potential conflict of interest.

## Publisher’s note

All claims expressed in this article are solely those of the authors and do not necessarily represent those of their affiliated organizations, or those of the publisher, the editors and the reviewers. Any product that may be evaluated in this article, or claim that may be made by its manufacturer, is not guaranteed or endorsed by the publisher.
